# Actinomyces europaeus Brain Abscess in a 69-Year-Old Female Causing Irreversible Neurologic Complications

**DOI:** 10.7759/cureus.42868

**Published:** 2023-08-02

**Authors:** Deesha R Bhojwani, Paragkumar Patel, Seung Ah Kang, Andres Bran

**Affiliations:** 1 Internal Medicine, University of Missouri School of Medicine, Columbia, USA; 2 Infectious Diseases, University of Missouri School of Medicine, Columbia, USA

**Keywords:** bacterial cns infection, mastoiditis, brain abscess, actinomycosis, actinomyces europaeus

## Abstract

Actinomycosis is a chronic, indolent, granulomatous disease process caused by the *Actinomyces* genus of bacteria. More severe forms of actinomycosis include disseminated or central nervous system (CNS) infections. *Actinomyces* *meyeri* is the most common species of *Actinomyces* isolated from brain abscesses. *A. europaeus* species is commonly associated with skin and soft tissue abscesses. However, it rarely causes brain abscesses. We present an unusual case of *A. europaeus* brain abscess in a 69-year-old female who presented with acute encephalopathy and bilateral lower extremity weakness. She was diagnosed with left-sided mastoiditis with intracranial extension, left posterior fossa epidural abscess, and transverse sinus thrombosis. The patient’s hospital course was complicated by hydrocephalus and declining neurological status. Empiric antimicrobial therapy was initiated, and the patient underwent mastoidectomy and external ventricular drain placement followed by decompression craniotomy and subarachnoid abscess aspiration. Given her poor and unchanged neurologic status, the patient was transitioned to comfort-oriented measures after shared decision-making with the family. It is crucial to identify *Actinomyces* as a causal agent of severe CNS infections like brain abscesses, meningoencephalitis, or subdural empyema, as untreated infections can lead to irreversible neurologic complications.

## Introduction

*Actinomyces* *europaeus* is a gram-positive, non-acid fast, non-motile, facultative anaerobic to microaerophilic rod that is a commensal found in the oral cavity, genitourinary tract, and gastrointestinal tract [1]. It was first identified by 16s rRNA gene sequencing in 1997 [2]. Most common manifestations of *A*. *europaeus* infections are skin and soft tissue infections like abscesses or necrotizing fasciitis, urinary tract infections, purulent urethritis, breast abscess, or decubitus ulcers/fistulizing disease [1,3,4].

## Case presentation

A 69-year-old female with an unremarkable medical history presented to an outside hospital with acute onset of confusion. Five days before her presentation, the patient suffered a mechanical fall due to weakness in the bilateral lower extremities. She had no history of loss of consciousness or head trauma. Patient's functional capacity had declined over the next few days along with a decrease in appetite. On the day of her presentation, she was confused and agitated. She was a current smoker with forty pack-year smoking history and did not use alcohol or recreational drugs. She did not have a recent history of swimming. The family noted that the patient had a 10-15 year history of left-sided ear pain with occasional discharge from the same ear for which she did not seek medical attention. 

Before presenting to our hospital, she became progressively more lethargic and was intubated for airway protection. On arrival, her vital signs were as follows: temperature: 36 degree Celsius; heart rate: 92 beats/min; blood pressure: 162/119 mmHg; and respiratory rate: 14 breaths/min. Her oxygen saturation was 99% on ventilation with 50% FiO_2_. Neurological examination (with sedation held) was pertinent for ability to follow commands and open her eyes to noxious stimuli; pupils were equal, round, and reactive to light and accommodation, diameter 3 mm bilaterally; gag reflex absent; moved all four extremities spontaneously, and Babinski sign was down-going bilaterally. Examination at the outside hospital noted several dental caries. The remainder of the physical exam was normal.

Initial laboratory studies were remarkable for marked leukocytosis with a left shift, mild hypokalemia, hyperglycemia, and mildly elevated transaminases (Table 1).

**Table 1:** Complete blood count, comprehensive metabolic panel, and other miscellaneous labs ANC: Absolute neutrophil count; ALT: alanine transaminase; AST: aspartate aminotransferase; BUN: blood urea nitrogen; CBC: complete blood count; CMP: complete metabolic panel; Hb: hemoglobin; Hct: hematocrit; Lab: laboratory; MCV: mean corpuscular volume; TSH: thyroid stimulating hormone; WBC: white blood cell

Lab test (unit)	Results day 1	Reference value
CBC		
Hb (g/dl)	14.4	12.0-16.0
Hct (%)	43.6	37.0-47.0
MCV (fl)	93.0	80.0-100.0
WBC (x 10^9/l) (thousand/mm3)	19.8	4.8-10.8
Neutrophils (%)	92.1	43.0-78.0
Lymphocytes (%)	2.6	20.0-40.0
Monocytes (%)	3.9	2.0-10.0
Eosinophils (%)	0.0	0.0-7.0
Basophils (%)	0.2	0.0-2.5
ANC (x 10^9/l) (thousand/mm3)	18.2	
Platelet count (x10^9/l) (thousand/mm3)	312	130-440
CMP		
Na+ (mmol/l)	139	136-145
K+ (mmol/l)	3.4	3.5-51
Cl- (mmol/l)	106	98-107
HCO3- (mmol/l)	26	21-32
Serum glucose (mg/dl)	171	70-100
BUN (mg/dl)	20	7-18
Creatinine (mg/dl)	0.61	0.51-0.95
Calcium (mg/dl)	9.7	8.3-10.6
Total protein (g/dl)	7.1	6.4-8.5
Albumin (g/dl)	3.0	3.4-5.0
Total bilirubin (mg/dl)	0.8	0.2-1.0
AST (units/L)	54	15-37
ALT (units/L)	64	10-49
Alkaline phosphatase (units/L)	165	45-117
Other miscellaneous labs		
Magnesium (mg/dl)	2.0	1.8-2.4
Ammonia (micromole/L)	<10	0-33
Blood alcohol g/dl (%)	0.000	0.000-0.000
Lactic acid (mmol/L)	1.10	0.40-2.00
TSH (uIU/ml)	0.365	0.358-3.740

**Figure 1:**
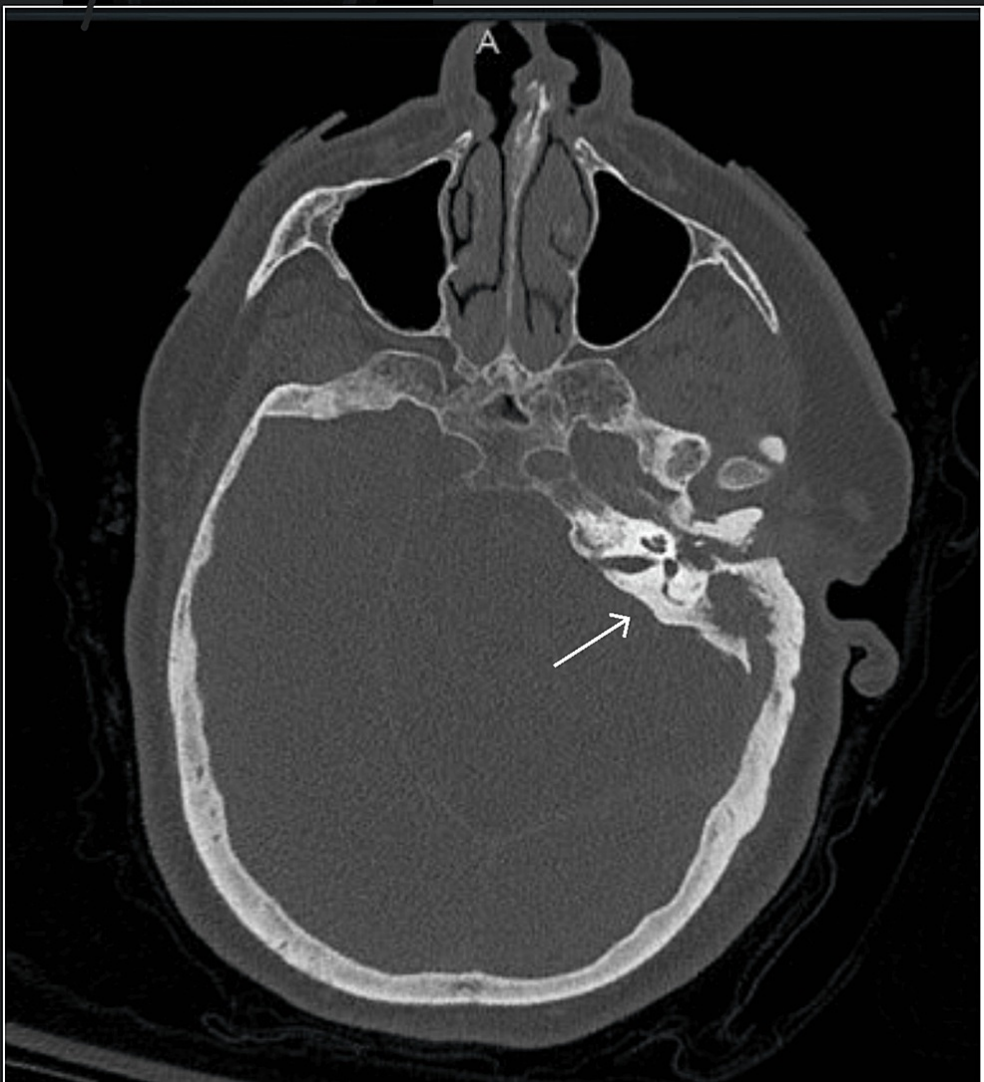
CT head revealing acute left-sided mastoiditis CT: Computed tomography

**Figure 2:**
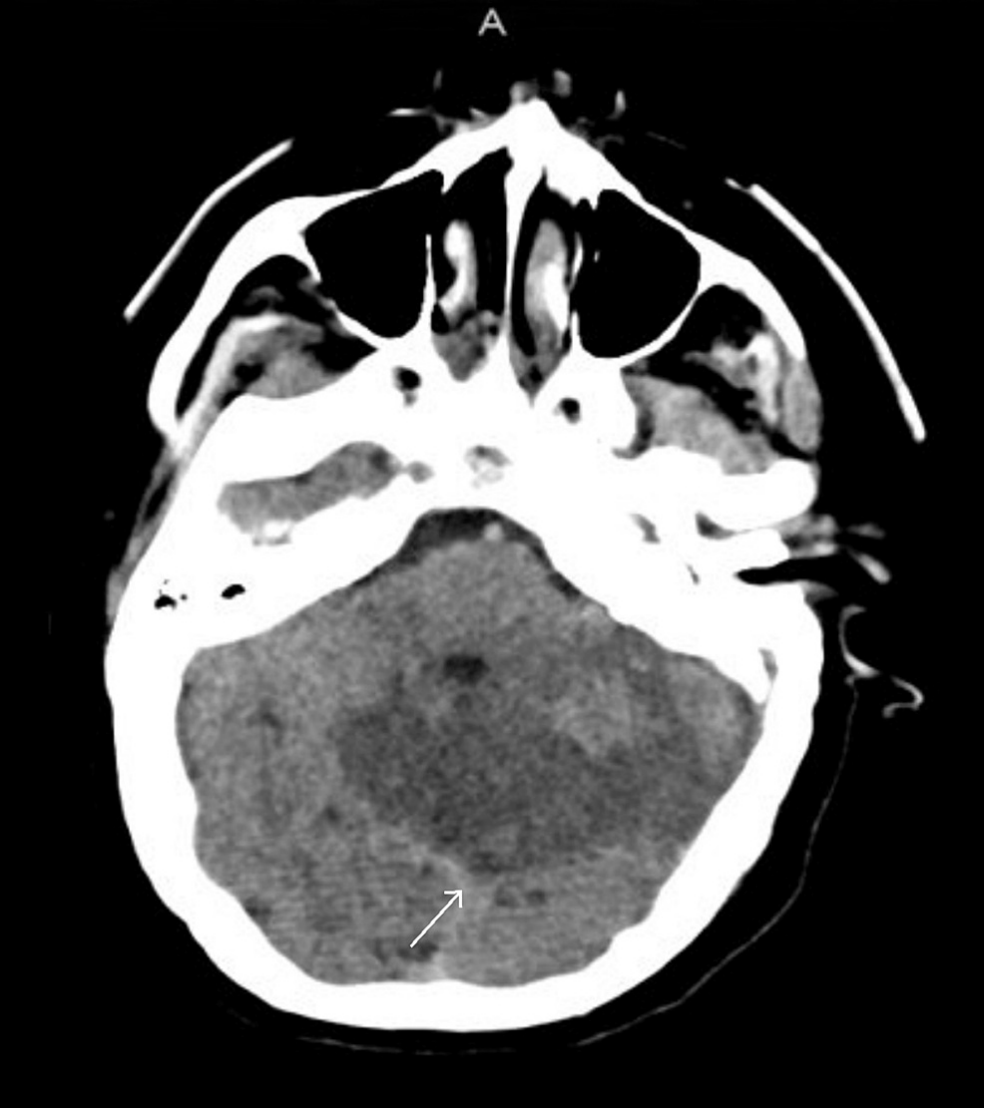
CT head showing left cerebellar infarct CT: Computed tomography

The urinalysis was normal, without evidence of infection. Urine drug screen and COVID-19 nasopharyngeal PCR test were negative. A computed tomography (CT) scan of the head and temporal bones showed acute left otomastoiditis with intracranial involvement, severe periodontal disease, and a subacute infarct of the left cerebellum (Figures 1, 2).

**Figure 3:**
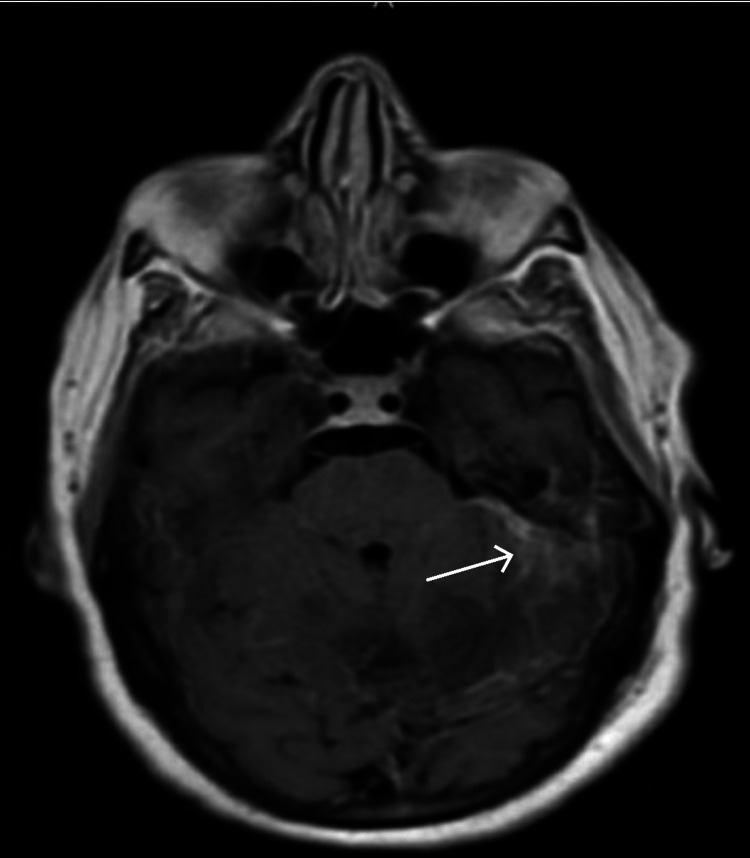
MRI brain showing left posterior fossa epidural abscess on T1 sequence MRI: Magnetic resonance imaging

**Figure 4:**
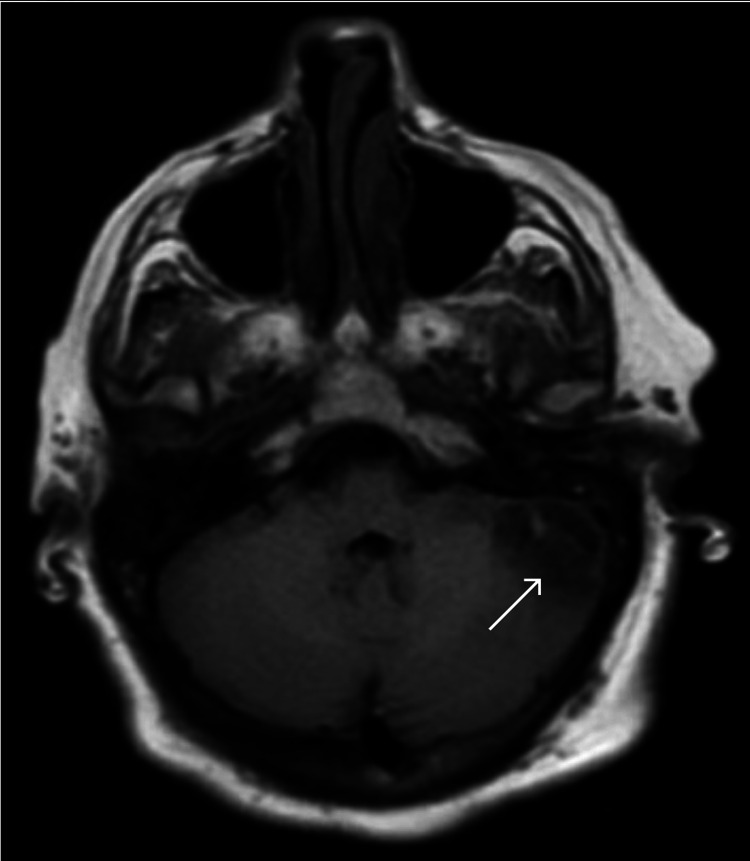
MRI brain showing left cerebellitis and posterior fossa epidural abscess on T1 sequence MRI: Magnetic resonance imaging

Further evaluation with magnetic resonance imaging (MRI) of the head demonstrated left mastoiditis with invasion into the intracranial space and left posterior fossa epidural abscess, cerebellitis, and a partially occluded left transverse sinus venous thrombosis (Figures 3, 4).

Low-intensity heparin was initiated due to concern for transverse sinus venous thrombosis. Neurosurgery, neurology stroke team, and otorhinolaryngology were consulted.

On day one of hospitalization, left-sided myringotomy and pressure equalizer tube insertion were performed, with mucopurulent fluid removal. The patient was given 10 mg of intravenous (IV) dexamethasone and was started on IV vancomycin and metronidazole. Due to her stable clinical examination, further neurosurgical procedures were held off with anticipation of a planned mastoidectomy. However, on day two, her neurological exam declined. The patient became comatose, and she had pinpoint and fixed pupils on the exam. A repeat CT head showed an evolving posterior cerebellar infarction with compression on the fourth ventricle resulting in acute hydrocephalus. Heparin was discontinued, and protamine was administered in anticipation of an external ventricular drain (EVD) placement for hydrocephalus. As the patient’s neurological exam did not improve after EVD placement, a left-sided decompressive posterior fossa craniotomy was performed, and subarachnoid pus collection was observed intraoperatively. The patient also underwent a mastoidectomy with frank purulence that was noted on the entrance to the mastoid antrum. Cultures were obtained from both procedures. Repeat CT and computed tomography angiography (CTA) and venography (CTV) of the head revealed improvement of the hydrocephalus status post-EVD placement, as well as subacute left cerebellar infarct and no evidence of significant intracranial arterial abnormalities or dural venous sinus thrombosis. IV ampicillin and cefepime were added to the antibiotic regimen on day three of hospitalization. There was no growth in urine or blood cultures. Cerebral spinal fluid (CSF) analysis performed on day four of hospitalization showed cloudy yellow fluid with a total cell count of 4,561/mm3 with predominantly segmented neutrophils, elevated protein, and low glucose, concerning for bacterial meningitis (Table 2).

**Table 2:** Cerebrospinal fluid analysis RBC: Red blood cell

Lab test (unit)	Results day 1	Reference values
Total volume (mL)	6.0 mL	
Glucose (mg/dL)	11mg/dl	40-75 mg/dl
Protein (mg/dl)	171mg/dl	15-45 mg/dl
Total nucleated cells (/mm3)	4561 /mm3	0-5 /mm3
Segs (%)	89 %	
Lymphocytes (%)	2 %	
Monocytes (%)	9 %	
Eosinophils (%)	0 %	
RBC (/mm3)	111/mm3	0
Clarity	Cloudy	Clear
Color	Yellow	Colorless

On hospital day five, an MRI brain showed ventriculitis, progression of the left cerebellar, and a new medial right cerebellar infarct along with new bilateral medial thalamic infarctions. The infectious disease (ID) team was consulted, and the regimen was changed to IV meropenem and continuation of IV vancomycin, with discontinuation of intravenous (IV) metronidazole, ampicillin, and cefepime. Serology for HIV and hepatitis C was negative. Cultures of the left mastoid left cerebellar epidural abscess grew *A*. *europaeus *on day seven and CSF cultures grew the same organism on day nine. Over the next few days, the patient’s neurological status remained unchanged. She continued to be comatose with inability to open her eyes and follow commands and had minimal withdrawal movements in all extremities. On hospital day seven, goals of care discussion was conducted with her family and it was decided to transition her to comfort care. The patient passed away on day 15.

## Discussion

The *Actinomyces* genus of bacteria is responsible for actinomycosis, a chronic granulomatous disease process leading to orocervicofacial, abdominopelvic, or thoracic infections. More severe forms of infection include disseminated and CNS infections [5]. Actinomycosis can happen in immunocompetent as well as immunocompromised patients. Risk factors include poor oral hygiene, dental procedures/infections, aspiration of oropharyngeal secretions for thoracic infections, invasive abdominal procedures/infections, intrauterine devices, and pelvic infections [5].

Proposed mechanisms for CNS spread off infections are direct spread from orocervicofacial actinomycosis, ear, and sinuses or hematogenous spread from a distant source, including thoracic or abdominopelvic actinomycosis [6]. CNS infections most commonly manifest as brain abscesses followed by meningitis or meningoencephalitis, actinomycetoma, subdural empyema, and spinal epidural abscess [6]. The common symptoms of brain abscesses are fever, fatigue, confusion, seizures, and neurological deficits [7]. Imaging modalities like CT scan with IV contrast or MRI aid in diagnosing *Actinomyces* brain abscess. They appear as peripherally enhancing lesions with a hyperintense rim on T1 weighted images on MRI. The lesions are usually encapsulated and can be single or multiple [7]. Diagnosis is confirmed by gram staining and histopathology, which show filamentous, gram-positive rods with necrosis and yellowish sulfur granules [8]. *Actinomyces* are indistinguishable from *Nocardia* on gram stain, but acid-fast stain helps identify the causative organism as the latter is acid-fast [7]. 

A 16s gene sequencing study on abscess specimens was carried out in Norway, revealing that Actinomyces species primarily seen in brain abscesses were *Actinomyces meyeri*, *Actinomyces georgiae*, and *Actinomyces israelii* [5]. Only one case has been described in the literature of *A*. *europaeus* as a cause of brain abscess [9]. 

The mainstay of therapy is beta-lactam antibiotics, preferably penicillin [10]. A prolonged duration of antimicrobial treatment, preferably six to twelve months, is recommended to treat actinomycosis to reduce the risk of recurrence [8]. Elimination of risk factors also helps to reduce the recurrence of actinomycosis. The only reported case of *A*. *europaeus* brain abscess is by Pan et al., which describes polymicrobial brain abscess secondary to acute on chronic suppurative otitis media in a 5-year-old male. Cultures from abscess grew *A*. *europaeus*, *Trueperella* *bernardiae*, and mixed anaerobes. The patient was treated with two stereotactic abscess aspirations and empiric IV antimicrobial therapy, followed by six months of amoxicillin monotherapy [9]. 

Corcione et al. reported a case of *A*. *europaeus* in a 21-year-old female with chronic progressive cervicofacial actinomycosis with vascular invasion secondary to a tympanic abscess. It was successfully treated with abscess drainage and IV ceftriaxone for one month, followed by oral amoxicillin for five months [10]. An* A.* *europaeus* case reported by White and Woodley described a breast abscess in a 69-year-old penicillin-allergic female who was successfully treated with abscess drainage and IV tigecycline, followed by oral clarithromycin [11]. 

## Conclusions

It is critical to identify *Actinomyces* as a causative agent of brain abscesses to direct appropriate antimicrobial therapy. Early identification can be challenging, especially with an insidious onset of symptoms compared to acute presentation. Delays in diagnosis can lead to catastrophic neurological complications, including brain infarction, cranial nerve paralysis, venous sinus thrombosis, hydrocephalus, and coma, as seen in our case.
